# IL-27 expression regulation and its effects on adaptive immunity against viruses

**DOI:** 10.3389/fimmu.2024.1395921

**Published:** 2024-06-20

**Authors:** Fernando Andres-Martin, Cooper James, Marta Catalfamo

**Affiliations:** Department of Microbiology Immunology, Georgetown University School of Medicine, Washington, DC, United States

**Keywords:** IL-27 (interleukin 27), CD8 T cells, IL-27 viral infection, IL-27 viral immunity, IL-27/IL-27R

## Abstract

IL-27, a member of the IL-6/IL-12 cytokine superfamily, is primarily secreted by antigen presenting cells, specifically by dendric cells, macrophages and B cells. IL-27 has antiviral activities and modulates both innate and adaptive immune responses against viruses. The role of IL-27 in the setting of viral infections is not well defined and both pro-inflammatory and anti-inflammatory functions have been described. Here, we discuss the latest advancements in the role of IL-27 in several viral infection models of human disease. We highlight important aspects of IL-27 expression regulation, the critical cell sources at different stages of the infection and their impact in cell mediated immunity. Lastly, we discuss the need to better define the antiviral and modulatory (pro-inflammatory vs anti-inflammatory) properties of IL-27 in the context of human chronic viral infections.

## Introduction

The interleukin 12 (IL-12) family of cytokines formed by IL-12, IL-23, IL-27, IL-35 and IL-39, plays critical roles in the induction and regulation of innate and adaptive immune responses (1). IL-12, IL-23 and IL-27 are secreted by antigen presenting cells including B cells, monocytes/macrophages and dendritic cells (1–4). IL-12 and IL-23 are pro-inflammatory and are involved in the generation of the T helper subsets, Th1 and Th17 respectively. Additionally, IL-12 is an important factor for NK cell activation and IFNγ secretion (5). In contrast, IL-35 is produced by regulatory T (Treg) and B (Breg) cells and therefore exerts important immunoregulatory functions (6–8). The new member of this family, IL-39, has been shown to be involved in the pathogenesis of murine experimental lupus erythematosus, however, its role in human disease is still under evaluation (9).

IL-27 is a member of this family and has been shown to promote both pro-inflammatory and anti-inflammatory functions. IL-27 induces the development of Th1 cells in response to bacterial and parasitic infections (10–13). In addition, it facilitates the development of T follicular helper (T_FH_) cells via the induction of IL-21, regulating B cell function (14). The regulatory functions of IL-27 include inhibition of Th2 and Th17 cell differentiation, and the induction of IL-10-producing Type 1 regulatory cells (Tr1) cells controlling immunopathology in the setting of infection (15–18).

In the setting of viral infections, the role of IL-27 is not well understood. IL-27 has antiviral properties against human viruses including influenza, herpes simplex, human hepatitis B (HBV) and C (HCV), human immunodeficiency virus (HIV) and others viruses, which underscores the therapeutic potential of this cytokine (19–30). The antiviral effects of IL-27 has been recently reviewed in detail by Amsden et al. (30). In the present manuscript, we will discuss the role of IL-27 in T cell mediated immunity in the setting of viral infection.

## IL-27/IL-27R signaling

**Figure 1:**
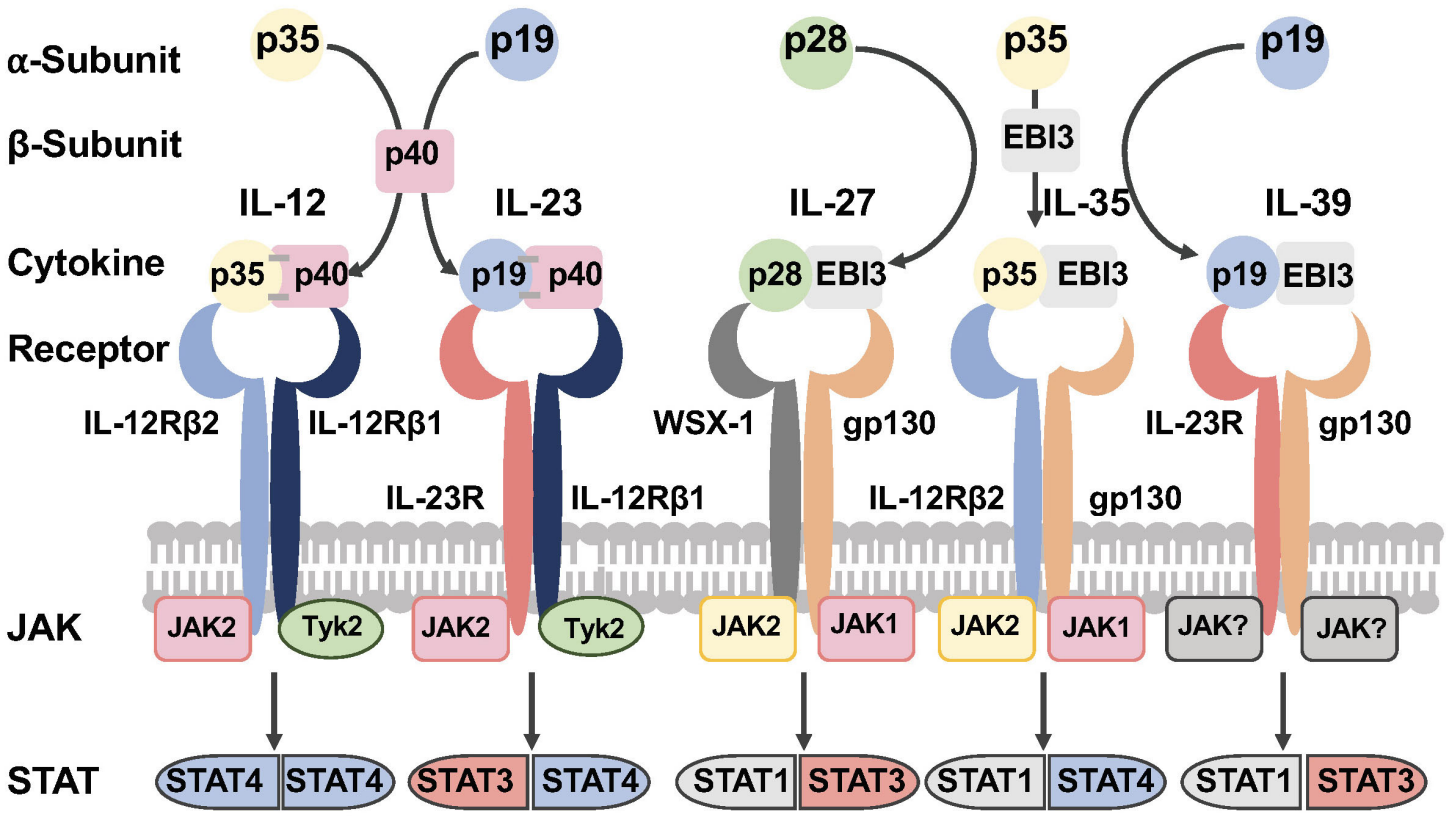
The IL-12 family of cytokines. The IL-12 family of cytokines are heterodimers that result from the combination of one of three alpha (α) chains (p19, p28 or p35), with one of two beta (β) chains (p40 or EBI3). The association of the α and β chains results in heterodimers that share a common element among the family members. The receptors of this family of cytokines are heterodimeric molecules shared by the family members. The cytokines of this family signal through the Janus Kinase (JAK)-Signal Transducer and Activator of Transcription (JAK/STAT) pathway (Figure was designed with BioRender.com).

The IL-12 family of cytokines are heterodimers that result from the combination of one of three alpha (α) chains (p19, p28 or p35), with one of two beta (β) chains (p40 or EBI3). The association of the α and β chains results in heterodimers that share a common element among the family members (**Figure 1**). IL-12 is composed by p35 and p40 subunits, and IL-23 results from the paring of p19 and p40 chains. IL-27 is formed by the association of p28 and EBI3. Lastly, EBI3 acts as the common element for IL-35 and IL-39 by pairing to p35 or p19 respectively (**Figure 1**) (10, 31–35).

A distinctive feature among the family members is that IL-12 and IL-23 are secreted as disulfide-linked heterodimers (10, 31, 36–40). In contrast, IL-27 and IL-35 are non-disulfide-linked, and it has been postulated this may impact the *in vivo* stability, resulting in lower secretion levels compared to disulfide-linked heterodimeric cytokines (10, 31, 40). The non-disulfide-linked features of IL-27 and IL-35 heterodimers present a challenge for their detection in tissues and measurements in the plasma.

The secretion of murine and human IL-27p28 shows interspecies differences. The folding and secretion of IL-27p28 depend on the formation of a disulfide-bond (Cys residue). In mice, the IL-27p28 subunit contains a single disulfide bond that stabilizes the protein and allows its independent secretion (10, 41–43). In contrast, the human IL-27p28 has only one cysteine residue and therefore lacks a disulfide-bond, instead requiring EBI3 binding for its secretion (10, 13, 43–46). The EBI3 subunit acts as chaperone-like protein and this property facilitates the secretion of non-disulfide-linked IL-27, IL-35, and IL-39 (45, 47).

In addition, computational studies of IL-27p28 Cys residue number and location across different species showed that in species with independent secretion of IL-27p28 such as mice, EBI3 is secretion incompetent and therefore retained inside the cells (43, 45, 48). This evolutionary feature of IL-27 has potential biological implications although it is not well understood and warrants further study.

IL-27 signals through a heterodimeric receptor composed of two subunits, the IL-27Rα (also known as WSX-1 or TCCR) and gp130 (IL6ST) (**Figure 1**) (10, 49, 50). The quaternary structure of IL-27 bound to its receptor resembles IL-6 and this structure reveals key interactions between IL-27Rα with IL-27p28 and the D2 domain of EBI3. In addition, a conserved tryptophan residue of IL-27p28 interacts with the D1 domain of gp130 (51, 52).

IL-27 binding activates the Janus Kinase (JAK)-Signal Transducer and Activator of Transcription (STAT1 and STAT3), and the mitogen activated protein kinase (MAPK) signaling pathway (**Figure 1**) (50, 53–59). IL-27 and IL-6 share a receptor subunit gp130 and both activate the transcription factor STAT3, however these cytokines exert different functions on target cells upon receptor binding. Studies in murine T cells using chromatin immunoprecipitation-sequencing in STAT deficient T cells showed that STAT3 plays an important role driving the overall transcriptome changes for both IL-27 and IL-6 (60). STAT1 was also found to be essential in providing the specific transcriptome normally induced by IL-27 signaling (60).

In human T cells, comparison of the global transcriptional changes induced by IL-27, IFNα and IL-6, showed that IL-27 clustered separately from IFNα and IL-6 signaling, but was closer to IL-6 than to IFNα. IL-27 induced a set of genes downstream of STAT1 that were also upregulated by IFNα and, to a lesser degree by IL-6 signaling (61).

## IL-27 expression in lymphoid and peripheral tissues

IL-27 is mainly produced by antigen presenting cells (APCs) including dendritic cells (DCs), plasma cells, macrophages, inflammatory monocytes, microglia, B cells and endothelial cells (62–66).

IL-27 mRNA expression showed high levels of EBI3 compared with IL-27p28 (10, 67). Similarly, *in vitro* experiments using LPS stimulation of monocyte-derived DCs showed that expression of IL-27p28 transcripts were transient compared to EBI3 which was more sustained (10). These observations suggest that the expression of heterodimeric IL-27p28 protein is regulated and highlights potential for other functions of EBI3.

The noncovalent nature of the IL-27 heterodimer and the independent secretion of the subunits had made it challenging to measure the bioactive heterodimeric form of IL-27 (10, 43, 45). In human tissues few studies have investigated the coexpression of both subunits to assess the main cell sources and expression dynamics in health and disease (**Table 1**). IL-27p28 protein expression was investigated in post-mortem human lymph nodes, and in lymph node biopsies from patients with granulomatous disease (sarcoidosis, tuberculosis and Crohn’s disease), which is associated with an epithelioid granuloma with accumulation of macrophages and CD4 Th1 cells (68, 69). IL-27p28 and EBI3 expression was observed CD68^+^ macrophages, blood and lymphatic endothelial cells and plasma cells (68, 69). EBI3 expression in lymph nodes was localized in follicles and inter-follicular areas, and in the T cell zone. In contrast IL-27p28 was expressed by plasma cells and fibroblasts, however coexpression of the subunits IL-27p28 and EBI3 was not observed in dendritic cells (69).

**Table 1:** IL-27 tissue expression in health and disease.

Specificity (reactivity)	Antibody Species	Cell Expression	Tissue Source	Disease
IL-27p28 (human and mouse)	Rabbit polyclonal	CD68+ macrophages but not in CD11c+ Dendritic cells Lymphatic sinuses and lymphatic microvessels	Human lymph nodes	Autopsies from individuals whose death was not associated with cancer, autoimmune, or inflammatory disease (68)
IL-27p28 (human)	Rabbit polyclonal antibodies directed against an N-terminal peptide of IL-27p28. Rat anti-IL-27p28 mAb (clone 29B5, Rat IgG2a)	Lymph nodes: Expression was observed in B cell follicles and intrafollicular. Macrophages, plasma cells and endothelial cells co-expressed both EBI3 and IL-27p28 subunits. Coexpression was not observed in dendritic cells. Lamina propria: coexpression of IL-27p28 and EBI3 and macrophages and endothelial cells. Partial overlap of IL-27 subunits was noted across the tissues.	Human lymph nodes, lamina propia	Biopsies of granulomatous disease sarcoidosis, tuberculosis and Crohn’s disease. Follicular hyperplasia from unknown origin (69)
EBI3 (human)	Mouse anti-EBI3 mAb (clone: 2G4H6, IgG2a)			
IL-27p28	Rabbit polyclonal Rat anti-IL-27p28 mAb (clone 29B5, Rat IgG2a)	Syncytiotrophoblasts and extravillous trophoblasts: coexpression of IL-27p28 and EBI3 IL-27p28 lower expression levels compared to EBI3 subunit	Human placenta (first, second and third trimester of pregnancy) and choriocarcinomas	Human placenta at distinct stages of pregnancy (67, 70)
EBI3 (human)	Mouse monoclonal anti-EBI3 Ab (clone 2G4H6)			
EBI3 (human)	Mouse monoclonal anti-EBI3 Ab (clone 2G4H6)	Human neutrophils in tissues	Tissue biopsies	Tissues of individuals affected by Gorham Disease, diverticulitis cholecystitis, and *Bartonella Henselae*infections showing suppurative lymphadenitis (71)
IL-27p28 (human)	Goat Polyclonal	Astrocytes, microglia and macrophages coexpressed IL-27 subunits. Partial overlap with EBI3 expression IL-27 subunits were highly expressed in individuals with Multiple Sclerosis (MS) compared to non-CNS disease	Brain	Post-mortem from patients with MS and donors without a CNS disease (72)
EBI3 (human)	Rabbit polyclonal			
EBI3 (human)	Monoclonal (2G4H6)	Astrocytes expression of EBI3 at the demyelinated region	Brain	Post-mortem from patients with MS and donors without a CNS disease (73)

Interestingly, IL-27p28 expression partially overlapped with EBI3 in macrophages and endothelial cells (69). The partial coexpression of IL-27 subunits in tissues is consistent with a transient production of *IL-27p28* mRNA transcripts observed in *in vitro* activated human DCs (10, 67). In addition, this observation may reflect the expression of other members of this family, IL-35 and IL-39, that share EBI3 (69).

IL-27 has also shown to modulate inflammatory responses in the brain (**Table 1**). IL-27 subunit expression was investigated in brain tissue obtained from individuals with Multiple Sclerosis (MS) and donors without clinical and neuropathological evidence of CNS disease (control samples) (72, 73). IL-27 subunits were mainly detected in astrocytes, microglia and macrophages; and their expression was increased in individuals with MS compared to the controls. Similar to the expression pattern reported in lymph nodes, in the brain, there was partial overlap of the IL-27 subunits coexpression (72, 73).

In tissues, IL-27 is produced by immune resident cell and cells recruited to the sites of inflammation (73–75). IL-27 signaling is involved in other pathological conditions such as atherosclerosis, non-small cell lung cancer (NSCLC) and other inflammatory diseases but the dynamics of IL-27 subunit coexpression in human tissues in the setting of health and viral infections remains scarce (76–80).

A common observation across tissue expression studies is the prominent expression of EBI3 and partial overlap with IL-27p28 subunits expression (**Table 1**). EBI3 is induced under inflammatory conditions and independently secreted, even in the absence of the subunits IL-27p28, IL-35p35 or IL-39p19. This has led to the hypothesis that EBI3 plays additional roles including a chaperone-like and regulatory functions (47, 67, 70, 71, 81, 82).

## IL-27 signaling in the context of viral infections

### Lymphocytic choriomeningitis virus

The murine model of infection with the Lymphocytic Choriomeningitis Virus clone 13 (LCMV Cl-13) establishes a chronic infection characterized by CD8 T cell immune exhaustion and high viral loads that mimics many features of human chronic viral infections including HIV, HBV, and HCV (83). LCMV-specific CD8 T cells are dysfunctional and express checkpoint receptors (84–88).

In this model, deficiency of IL-27 signaling (IL-27RA^-/-^ mice) leads to higher viral titers and uncontrolled lifelong viremia compared to the wild type mice (89, 90). In the spleen of the infected mice, detectable *IL27p28* mRNA transcripts were found in DCs subsets including plasmacytoid DCs (pDCs) and macrophages very early post infection (day 1 p.i) and returned to basal levels at day 8 p.i (89). In contrast, no changes were observed in *Ebi3* mRNA transcripts were observed during infection, with only a slight reduction at a later time point (89). The rapid upregulation of IL-27 expression (IL-27p28) occurs with the early induction of Type I IFNs (91, 92). Indeed, IL-27RA deficient mice showed reduced Type I IFN production in both acute and chronic LCMV infection (89).

The T cell mediated responses in the absence of IL-27 signaling showed a significant early expansion of LCMVGp33-specific CD8 T cells (day 9 p.i) compared to the wild type mice, and returned to similar levels at day 30 p.i. Interestingly, despite lower expression of PD1 (a marker of exhaustion LCMV chronic infection), virus-specific CD8 T cells demonstrated similar ability to secrete cytokines to wild type T cells (89). Additionally, IL-27 signaling promoted survival of virus-specific CD4 T cells and viral control during early infection (89, 92, 93).

Moreover, IL-27 signaling is an important factor for a subset of exhausted memory CD8 T cells with stem-like properties (CXCR5^+^) that confers antiviral immunity during chronic LCMV infection (92, 94, 95). In these elegant studies, the authors showed that treatment with IL-27 significantly increased virus-specific CXCR5^+^CD8 T cells and reduced viral titers in the brain (92). IL-27 signaling was critical for CXCR5^+^CD8 T cell expansion, and this effect was in part mediated by STAT1-driven IRF1 expression preventing terminal differentiation and cell death.

In this model, B cell-derived IL-27 is a crucial factor for the survival and function of virus-specific CD4 T cells and T_FH_ cells, enhancing humoral immunity and viral control at the later phase of the infection (89, 96). Altogether, these studies showed that in chronic LCMV infection, IL-27 is beneficial and plays an essential role in both T cell and humoral immunity.

### Cytomegalovirus infection

The human cytomegalovirus (HCMV) infection establishes latency and persist at mucosal sites, including the salivary glands. In human and murine CMV infection, CD4 T cells are key players in the control of viral replication in the salivary glands (97, 98). Particularly, memory T cells control the virus in periods of viral reactivation preventing development of clinical disease (99). HCMV evades the immune system by modulating host responses including the induction IL-10 resulting in viral persistence (100–102). In addition, CMV infection has been associated with the expansion of virus-specific CD8 T cells with a senescent phenotype (103, 104).

The role of IL-27 was investigated in a murine model of CMV infection (MCMV). The studies showed that early in infection (2 days p.i), IL-27p28 is expressed in the spleen by myeloid cells (DCs, pDCs, macrophages and neutrophils) and B cells (98, 105). In contrast, in the salivary glands, its expression is detected during the viral persistence phase (day 14 p.i) (105). Induction of IL-27 was dependent on Type I IFN, as *in vivo* blockade of IFNAR reduced IL-27 expression by DCs, macrophages B cells and neutrophils (105).

IL-27 signaling regulated T cell mediated immunity by enhancing the expansion of virus-specific CD4 T cells including those that provide immunity during viral persistence. In contrast, these effects were not observed in CD8 T cells, which may be due to the lower IL-27Rα expression on CD8 T cells when compared with CD4 T cells (98). In the salivary glands, IL-27 signaling promoted virus-specific CD4 T cells that secrete IFNγ and IL-10 and resembled Tr1 cells, which contributed to viral persistence (98, 105–109).

In human CMV infection CD4 and CD8 virus-specific T cells play a critical role in controlling CMV, however whether IL-27 contributes to viral persistence is not well understood (110). Human CMV-specific CD4 T cells have a cytotoxic phenotype and express CX3CR1 suggesting a potential contribution to chronic endothelial inflammation and injury (111–115). Similar to the murine model, human CMVpp65 and CMVgb -specific CD4 T cells from peripheral blood and colon produce IL-10, although variable frequencies were present among individuals (105). The role of IL-27 in CMV infection requires further investigation to fully determine the modulatory effects in the context of CMV infection.

### SIV infection

IL-27 has *in vitro* antiviral properties against HIV and other human viruses reviewed elsewhere (21, 22, 27, 30, 116, 117). Limited data of the coexpression of IL-27 subunits and kinetics of expression is available in the setting of SIV/HIV infection. In non-human primates infected with Simian Immunodeficiency Virus (SIV), interferon-stimulated genes were evaluated in the spleens of animals before and after infection at days 4, 7, 14, 21, and 56 p.i (118).

*IL27p28* mRNA expression was induced in the acute phase similarly to *IFNB* transcripts, and both remained high compared to the uninfected animals during the chronic phase of infection (>56 days p.i). In addition, *IL10* mRNA transcripts were also detected in the acute phase, however its expression was reduced to basal levels during the chronic phase (118).

The role of IL-27 in CD4 T_FH_ cell differentiation was investigated in SIV infected rhesus macaques (RM) (119). This study evaluated mRNA of the IL-12 family subunits (*p19*, *p40*, *p35*, *p28* and *EBI3*) in cell suspensions of mesenteric lymph nodes from SIV infected RM with high and low CD4 counts. mRNA transcripts of the subunits that form the cytokines IL-12, IL-23, and IL-35 were reduced in the infected animals with low CD4 counts. In contrast, no significant changes were observed for the expression of *IL27p28* and *EBI3* mRNA in the animals, irrespective of CD4 count or infection status (119).

In *in vitro* cultures of mesenteric lymph node cell suspensions, IL-27 induced downregulation of CXCR5 expression in CD4 T_FH_ (CXCR5^high^PD1^high^) and increased the frequency of cells expressing CXCR5^low^PD1^high^, where upregulation of Tbet expression and Th1-like function. This suggests that IL-27 may alter T_FH_ differentiation during mucosal immune response against SIV infection (119).

### HIV infection

There is no available data about IL-27 expression in tissues from people with HIV (PWH). Most reported studies have investigated plasma levels of IL-27 at distinct stages of the infection with no consistent conclusions about the potential *in vivo* regulation of IL-27 during HIV infection. A study reported no changes in circulating levels of IL-27 at several stages of infection, untreated, successfully suppressed viremia with ART (120). However, contradicting these results, another study showed IL-27 plasma levels were inversely correlated with viremia in naïve HIV monoinfected compared to HCV coinfected groups (121, 122). In PWH and CMV coinfection, IL-27 plasma levels were negatively correlated with viral load and positively associated with CD4 T cell counts suggesting a beneficial effect in CD4 T cell reconstitution in CMV infected PWH (123). Further, another study described a positive association between IL-27 plasma levels and provirus HIV-DNA in Peripheral Blood Mononuclear Cells (PBMCs) (124).

The effects of IL-27 in T cell immunity against HIV has been investigated in individuals with uncontrolled viral replication (125). In this report, Tregs were shown to secrete IL-27 inducing IL-10 production by monocytes, which resulted in blunted proliferation of HIV-specific CD4 T cells *in vitro*, although these effects were heterogenous among the individuals (125).

In the context of viral suppression by antiretroviral therapy (ART), T cells express higher levels of STAT1 which leads to enhanced STAT1 activation by IL-27 stimulation. This effect resulted in upregulation of T-bet expression by TIGIT^+^HIVGag-specific T cells and increased cytokine secretion and cytotoxic potential (expression of CD107a) (61). These results suggest that IL-27 can modulate the function of exhausted T cells during chronic HIV infection.

Moreover, in PWH and CMV coinfection, *in vitro* stimulation of PBMCs with CMVpp65 in the presence of IL-27 stimulation led to increased IFNγ secretion by CMVpp65-specific CD4 T cells in both CMV^+^PWH and CMV^+^PWOH (People Without HIV) (113, 123). IL-27 induced IL-10 secretion by IFNγ^+^CMV-specific CD4 T cells recapitulating Tr1 cells, but did not have an impact on the expansion of Tregs (CD25^+^FoxP3^+^) (105, 109, 123, 126).

### Respiratory viruses

Respiratory infections are diverse in terms of the viral agent, severity of the disease and contribution to morbidity and mortality worldwide. Herein, we discuss those in which IL-27 has been reported to play a role.

### Influenza

Seasonal influenza infection is a significant contributor of morbidity and mortality worldwide in young children and older adults (127). Influenza virus infection can drive massive pulmonary immune infiltration and immunopathology. In this setting, IL-10 plays an important role in regulating pro-inflammatory function of innate and adaptive cells to control immunopathology in the tissues (128).

Infection with a highly pathogenetic influenza strain in mice drove upregulation of the *Il27p28* mRNA subunit and *Il10* mRNA with similar kinetics in the lung that peaked at day 7 p.i. In contrast, *Ebi3* transcripts were detected at baseline and no changes were observed through the course infection. *Il27* mRNA subunits were highly expressed in the lungs compared to the spleens and the lymph nodes, and correlated with the lower viral load at day 7 p.i highlighting its potential antiviral effects (129).

IL-27 plays a critical role in limiting immunopathology by controlling neutrophil accumulation and reducing the inflammatory Th1 and Th17 cells by both IL-10-dependent and -independent mechanisms (130–133). IL-27 signaling promoted IL-10 production in IFNγ secreting virus-specific CD4 T cells and cytotoxic CD8 T cells (133).

In addition, the administration of IL-27 showed different effects, at the peak of the viral load (between 5–10 days p.i) it was beneficial and ameliorated the immunopathology by reducing the influx of pro-inflammatory cells into the lungs without impairing viral clearance (129). In contrast, when IL-27 was administered at early time point of the infection, 1–7 days p.i, disease worsened, albeit reducing immunopathology (129).

### Murine parainfluenza Sendai virus

Respiratory paramyxoviruses are important causes of morbidity and mortality, particularly of infants and the elderly. Acute murine parainfluenza virus Sendai (SeV) infection is followed by a chronic type 2 immune pathology in the lung similar to that observed in humans (134). mRNA *IL-27p28* transcripts are increased in the lungs during infection and IL-27 signaling promoted the generation of IL-10 secreting virus-specific CD8 T cells (135, 136). In addition, IL-27 promoted the development of IL-10 producing antiviral CD4 T cells resulting in the inhibition of the development of Th2 responses that mediates lung pathology during the chronic phase (137).

### Human influenza infection

Limited data is available on dynamics of IL-27 in the setting of human influenza infection, IL-27 serum levels were reported to be increased in individuals infected with seasonal IAV (H3N2) and H1N1. The cellular sources IL-27 have been mainly attributed to APCs, however, lung epithelial cells *in vitro* produce IL-27 after infection with influenza A through a COX-2-derived prostaglandin E2 (PGE2) mechanism. Accordantly, serum levels of IL-27 in individuals infected with 2009 pandemic H1N1, and with seasonal H3N2 were positively correlated with PGE2 (30, 138).

### SARS-CoV-2 infection

Severe acute respiratory syndrome coronavirus 2 (SARS-CoV-2) is a new strain of coronavirus that was first reported in December 2019. SARSCoV2 is the cause of the Coronavirus disease 2019 (COVID-19). The pathogenesis of severe disease is associated with an altered activation and regulation of the Type I IFN responses and an exacerbated inflammatory response, which contributes to the lung injury (139–142).

In individuals with COVID-19 disease, increased serum levels of IL-27 have been observed compared to healthy controls. In addition, lower serum levels were associated with severe disease in hospitalized patients in intensive care (143). Another study investigated levels of inflammatory cytokines and their potential association with lung damage and mortality at the time of hospital admission in 108 individuals. This study reported that low serum levels of IL-27 and higher levels of IL-1α and HGF were associated with poor clinical outcomes (144). Contrasting these observations, increased IL-27 subunit (*p28* and *EBI3*) mRNA expression was observed in PBMCs and monocytes from individuals with severe disease (145). All of these findings suggest that the upregulation of IL-27 may contribute to antiviral immunity against SARS-CoV-2 by inducing interferon stimulated genes. However, whether IL-27 production is beneficial or detrimental to the progression to severe COVID-19 remains unclear.

### Neurotropic mouse hepatitis virus (JHM strain)

Limiting immunopathology during central nervous system CNS viral infections is critical for the host survival. The murine hepatitis virus (JHMV) has a glia tropism and induces an acute demyelinating encephalomyelitis that leads to viral persistence and chronic demyelination (146). While local IL-10 production by infiltrating T cells limits tissue pathology, it also delayed viral control and elimination. IL-10^+^virus-specific CD8 T cells were highly activated, produce inflammatory cytokines, and have cytotoxic function suggesting that IL-10 may be a mechanism by which CD8 T cells regulate their own function at sites of tissue injury (147).

Mice deficient in the EBI3 subunit (shared by IL-27 and IL-35) and infected with JHMV showed immune mediated inflammation in the CNS. Accumulation of IFNγ secreting virus-specific CD4 and CD8 T cells lead to tissue damage and reduced survival. Interestingly, despite increased IFN-γ secretion by virus specific CD8 T cells the cytotoxic function was reduced (148). These studies imply that IL-27 and IL-35 may play a role in controlling immunopathology and disease severity by preventing recruitment of effector CD8 T cells.

In addition, IL-27 induces IL-10 production by virus specific CD4 but not CD8 T cells. IL-27R deficiency led to accumulation of IFN-γ secreting virus-specific CD4 and CD8 T cells and reduced IL-10 production by virus-specific CD4 T cells promoting viral control. In contrast, IL-10 production and cytolytic activity of virus-specific CD8 T cells was not reduced nor was there an effect on Tregs. The lack of IL-27 signaling did not enhance viral clearance in the CNS although the mice exhibited less severity of disease (149). These studies contrast with the protective role of IL-27 in limiting tissue pathology.

## Discussion

**Figure 2:**
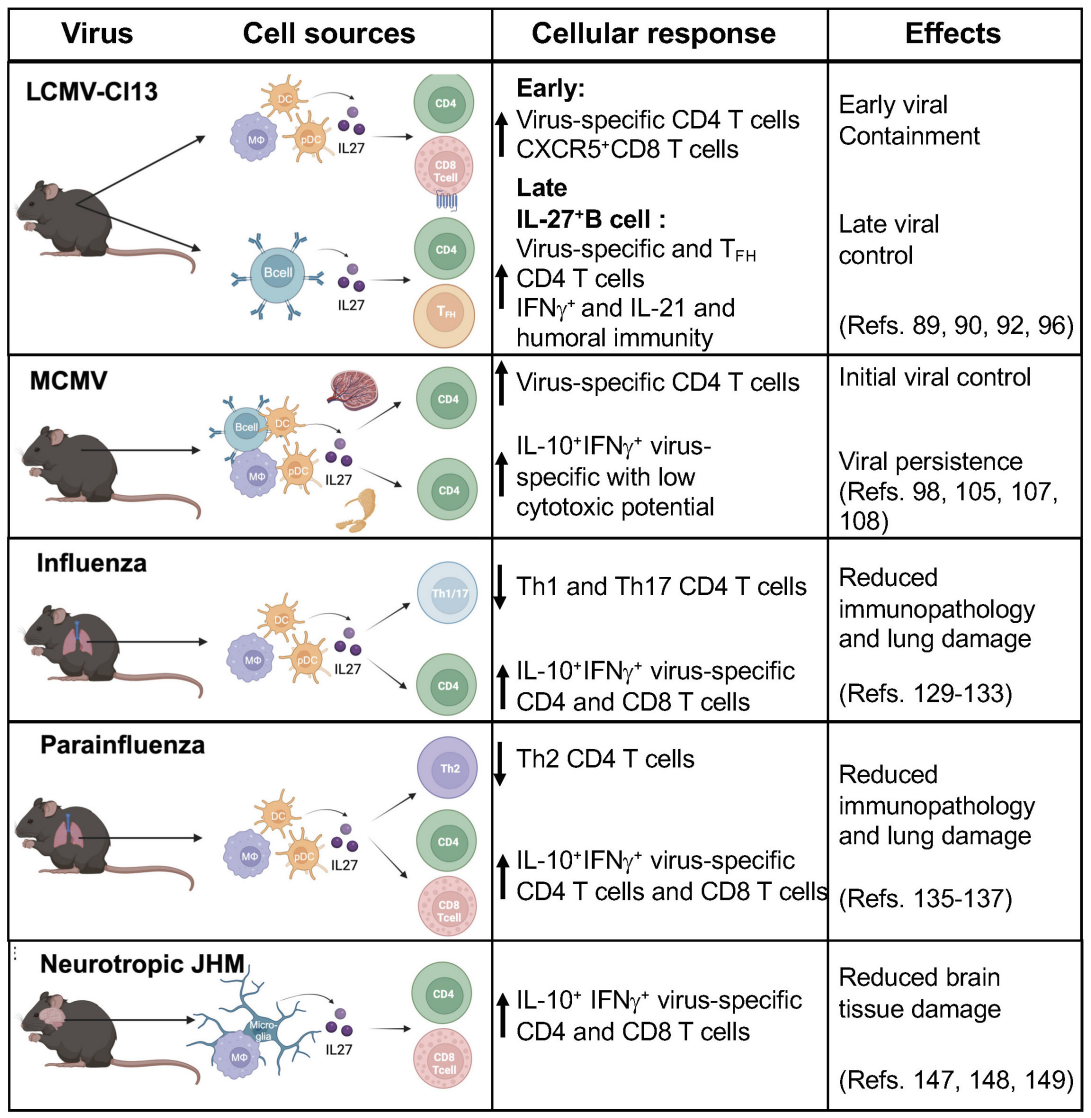
IL-27 cell sources and cellular responses during viral infection. IL-27 has antiviral activities that are prominent early after infection. IL-27 is mainly produced by antigen presenting cells (APCs) including dendritic cells (DCs), plasma cells, macrophages, monocytes, microglia, B cells and endothelial cells. IL-27 plays critical role by modulating adaptive cellular responses against viruses. The beneficial effects include reducing immunopathology and viral control, in contrast in some infections showed a predominant regulatory function leading to viral persistence. (Figure was created with BioRender.com).

IL-27 antiviral properties against several human viruses have highlighted its potential therapeutic use for the treatment of human viral infections (27, 30). However, how IL-27 mediates antiviral immunity leading to viral clearance and persistence is not well understood (**Figure 2**).

The cell sources of IL-27 in human lymph nodes in health and disease, showed that IL-27p28 and EBI3 subunits are coexpressed by macrophages, monocytes, B cells and endothelial cells, however IL-27p28 was not expressed in DCs (68, 69). One consistent observation in lymph nodes and other tissues is the high expression of EBI3 compared to IL-27p28 (**Table 1**). This has led to the hypothesis that EBI3 may play additional roles including chaperone-like function and/or regulatory functions (47, 67, 70, 71, 81, 82). In humans, *in vitro* studies showed that IL-27 can be produced by CD4 T_FH_ cells, in the setting of chronic HBV infection, and by Tregs in untreated HIV infection and uninfected controls (125, 150).

More importantly, murine *in vivo* studies using chronic LCMV infection have revealed important insights to its temporal expression, cell sources and impact in mediating innate and adaptive immune responses. During chronic LCMV infection, DCs and myeloid cells are the predominant sources of IL-27, mediating early viral control. Where lack of IL-27 signaling during this stage of infection leads to higher viral titers compared with wild type animals. IL-27 promotes expansion of virus-specific CXCR5^+^CD8 T cell and virus-specific CD4 T cell function. In contrast, IL-27 secreted by B cells was relevant at later phase of the infection and facilitated maintenance and function of virus-specific CD4 T cells as well as CD4 T_FH_ function, which promoted humoral immunity and viral clearance (89, 92, 96).

The regulatory functions of IL-27 in the setting of viral infection involves both IL-10 dependent and independent mechanisms. IL-27 induces IL-10^+^ secretion by IFNγ^+^virus-specific T cells (Tr1-like cells), controlling exacerbated inflammation and conferring host protection from immunopathology in the lung during influenza infection (129, 133). In contrast, in the setting of MCMV, expression of IL-27 and induction of IL-10 by Tr1-like cells contributed to viral persistence in the latent phase of the infection (98, 105).

In the context of human infections, the role of IL-27 is not well defined and studies of the kinetics of IL-27 expression *in vitro* and *in vivo* studies are limited. The detection of IL-27 in serum and plasma showed contradictory results, likely because the measurements of circulating levels of IL-27 has been challenging due to the non-disulfide-linked nature of IL-27 (IL-27p28 and EBI3) (31). Some ELISAs use polyclonal capture antibodies and reported cross-reactivity with recombinant EIB3, given there are potentially other cytokines that express this subunit this data is confounding (41, 46, 151, 152). More reliable tools to detect the heterodimer may allow us better understand its role in human viral infections.

IL-27 may have a potential clinical applications in the setting of viral infections, while its antiviral effects seem broad and synergistic with Type I IFNs, its modulatory effects require further investigation in the setting of viral infections. Current clinical applications of targeting IL-27 are more advanced in the setting of cancer and focus on blocking immunosuppressive activities (49, 153). A human monoclonal (IgG1) antibody against the IL-27p28 subunit CHS388 (former SRF388), that blocks IL-27/IL-27RA interaction is under consideration for cancer treatment, where a Phase I study has already been shown to be safe in humans (1, 154–158). However other studies, focus on the anti-tumor activities of IL-27 (159, 160).

IL-27 signaling modulates several immune cells and there is a need to better define its temporal expression and its cell sources during viral infections. It will be important to fill this gap in knowledge to dissect IL-27’s antiviral effects and modulatory (regulatory vs stimulatory) functions. Determining the outcome of this balance will be beneficial in the setting of human chronic infections including HIV, HBC, HCV and others.

## Author contributions

MC: Writing – review & editing, Writing – original draft. FA-M: Writing – original draft. CJ: Visualization, Writing – review & editing.
